# Vaginal delivery in women with perianal Crohn's disease: why not?

**DOI:** 10.1016/j.xagr.2024.100333

**Published:** 2024-03-14

**Authors:** Irene J. Schaafsma, Froukje J. Hoogenboom, Gerard Dijkstra, Jelmer R. Prins, Marijn C. Visschedijk

**Affiliations:** 1 Department of Gastroenterology and Hepatology, University of Groningen, University Medical Center Groningen, Groningen, the Netherlands (Drs Schaafsma, Dijkstra and Visschedijk); 2 Department of Surgery, University of Groningen, University Medical Center Groningen, Groningen, the Netherlands (Drs Schaafsma and Hoogenboom); 3 Department of Obstetrics and Gynecology, University of Groningen, University Medical Center Groningen, Groningen, the Netherlands (Dr Prins).

**Keywords:** Crohn's disease, delivery method, fecal incontinence, perianal disease, perianal disease progression

## Abstract

**BACKGROUND:**

Pregnant women with active perianal Crohn's disease indicate a cesarean delivery according to the current European Crohn's and Colitis Organisation guidelines. This advice is based on the assumption that vaginal delivery leads to exacerbation of perianal disease and worsening of fecal continence. However, there is no strong evidence to support this.

**OBJECTIVE:**

This study aims to examine the effects of the delivery method on perianal disease progression and fecal incontinence in women with perianal Crohn's disease.

**STUDY DESIGN:**

In this retrospective cohort study, 102 women were selected from the 1000 inflammatory bowel disease cohort of a tertiary hospital in the Netherlands. All women are aged >18 years, have perianal Crohn's disease, and have given birth. In addition, all women completed a questionnaire. Fecal continence was scored using the Vaizey score. Using SPSS, descriptive analysis and linear regression analysis were performed, and *P* values <.05 were considered statistically significant.

**RESULTS:**

The cesarean delivery rate within our cohort was 19.5%. Within the group of women who delivered at least one child vaginally (n=84), 25.5% reported alteration of fecal continence, compared with 13.1% in women who only had cesarean delivery (n=18). After a mean follow-up of 15 years, the median Vaizey score within the cesarean delivery group was 5, compared with 7 in the vaginal delivery group. Within the vaginal delivery group, 18.8% reported perianal disease progression, compared with 22.2% in the cesarean delivery group. No significant relation between mode of delivery and fecal continence or perianal disease progression was found (B, 0,97 [−1,19 to 3,14], *P*=.38).

**CONCLUSION:**

Fecal incontinence and perianal disease progression after vaginal delivery in Crohn's disease women with active perianal fistula is not significantly increased in this retrospective cohort. This study opens the discussion for more tailored obstetric advice in women with perianal Crohn's disease.

## Highlights

• Current guidelines on delivery methods in women with perianal Crohn's disease (CD), state that a cesarean delivery is preferred in women with active fistulizing perianal disease.
• In this study, a low cesarean delivery rate is seen within the group of women with perianal CD, and remarkably no significant difference is found in fecal incontinence or perianal disease progression in women who delivered vaginally than women who underwent cesarean delivery.
• This study supports the possibility of vaginal delivery in patients with perianal CD.

**AJOG Global Reports at a Glance**

**Why was this study conducted?**

Current guidelines state that women with active perianal Crohn's disease should deliver through a cesarean delivery. The reason for conducting this research was the question of what the outcome of a vaginal delivery would be in this patient group considering fecal incontinence and perianal disease progression.

**Key findings**

In this study, a low cesarean delivery rate is seen within the group of women with perianal Crohn's disease, and remarkably no significant difference is found in fecal incontinence or perianal disease progression in women who delivered vaginally than women who underwent cesarean delivery.

**What does this add to what is known?**

Based on this study, it could be debated that women with perianal Crohn's disease can deliver vaginally without a significantly higher risk of worsening fecal incontinence or progression of the perianal disease.

## Introduction

Crohn's disease (CD) is a chronic inflammatory bowel disorder and is mainly diagnosed within the reproductive years, as the peak incidence of CD is between 15 and 35 years of age.^1^ It has been shown that around 23% of the women with CD will develop perianal disease within 20 years after initial diagnosis. This percentage is elevated to 92% in the case of proctitis.^2^ It is estimated that around 25% of the women with CD will become pregnant after they have received the initial diagnosis.^1^

The European Crohn's and Colitis Organisation (ECCO) formulated guidelines in 2022 on reproduction and pregnancy in inflammatory bowel disease (IBD).^3^ In these guidelines, it is stated that the decision on the mode of delivery in women with perianal CD should always be subject to a multidisciplinary approach and that it should mainly be governed by obstetric indications. An example of an obstetric indication for a cesarean delivery is fetal distress. Besides any obstetric indications, active perianal disease is an indication for a cesarean delivery according to the ECCO guidelines. This statement is based on 2 studies that report perianal disease exacerbation after vaginal delivery in women with active perianal disease before delivery.^4^^,^^5^ In addition, a large retrospective study showed that preexistent perianal disease is associated with large perineal lacerations.^6^ It is important to avoid perianal damage as this might cause nonhealing wounds, fistulization, and fecal incontinence. However, the authors could not directly compare the mode of delivery, as there was not enough data available.

Various guidelines, besides the ECCO guidelines, state that a cesarean delivery is recommended over vaginal delivery to reduce the risk of perianal injury in women with CD who have active perianal disease.^7^, ^8^, ^9^ Therefore, it seems that there is international consensus on the advised mode of delivery in women with IBD and active perianal disease; however, these guidelines also state that they are based on limited evidence and multidisciplinary expert opinions.

An important aspect to take into account when deciding on the mode of delivery is the risk of worsening fecal continence in women with CD. This is especially relevant in women who already have risk factors for fecal incontinence, such as proctitis, high stool frequency, and earlier bowel resections. Previous studies in small patient groups showed contradictory results, suggesting worsening of the fecal continence because of vaginal delivery.^10^, ^11^, ^12^ Interestingly, a few studies have also shown that cesarean delivery is not protective when considering the amount of perianal disease progression and perianal damage.^13^^,^^14^ In literature, the cesarean delivery rates are high in women with perianal CD. Burke et al^15^ have shown that 83,3% of the women with the perianal disease in their medical history had a cesarean delivery, which is significantly higher than the reported 13,6% section rate in the general population in the Netherlands.^16^^,^^17^ 62,5% of the scheduled cesarean deliveries were due to an inactive perianal CD, whereas the ECCO guidelines state that only an active perianal CD is an absolute indication for a cesarean delivery.^3^

Current guidelines regarding the preferred mode of delivery in women with perianal CD are based on limited evidence and give limited direction for counseling without consideration of the location and complexity of the fistula. In addition, the distinction between an active and an inactive fistula is used, which can be a difficult distinction in daily practice. This retrospective cohort study aimed to investigate the effect of the mode of delivery on perianal disease and fecal continence in women with perianal CD.

## Materials and Methods

### Patients

Patients in the 1000 IBD cohort of University Medical Centre Groningen (UMCG), a tertiary referral hospital in the Netherlands, were retrospectively studied. All IBD patients aged ≥ 18 years who consented to the prospective Parelsnoer IBD biobank, approved by the UMCG medical ethical committee (reference METC 08/279), were included. The 1000 IBD cohort is an extensively structured database with IBD patients and is based on the Parelsnoer Institute. The Parelsnoer Institute is a collaboration between 8 Dutch University Medical Centers in which clinical data and biomaterials from patients suffering from chronic diseases (so-called “Pearls”) are collected according to harmonized protocols. This database was started in 2010. All 440 women with CD and perianal disease are included in this cohort and are extensively phenotyped. They gave informed consent to use the phenotype data. All data were registered by the gastroenterologist on every patient visit. The obstetric information was mostly added to the database, and we reviewed all data case by case.

The 440 female patients who were included were all diagnosed with CD and perianal disease in their medical records. No further characteristics were used as criteria at this point of inclusion of the patients. Within this group, 240 women (aged 25–85 years) have given birth to at least 1 child. The advice on the mode of delivery was given in a multidisciplinary manner to a gastroenterologist, gynecologist, and gastrointestinal surgeon and was recorded in the electronic patient file.

### Data analysis

The information extracted from the IBD database included the mode of delivery, gestational age, and birthweight. Furthermore, all patients were analyzed using information from the Electronic Patient Data System of the UMCG. A systematic approach was used to analyze each patient, including analysis of pelvic magnetic resonance images (MRIs). If the mode of delivery was a cesarean delivery, the medical indication and whether it was planned or emergency was reported. If the child was delivered vaginally, it was noted whether it was an assisted vaginal delivery (including vacuum-, forceps- and other instrumental extraction) and whether the patient had an episiotomy or a perineal laceration. For the perianal fistulas, it was noted whether the fistula was present during delivery, whether it was inactive or active, and what the location was. Women were only included if the presence of perianal fistulas was reported by the gastroenterologist on outpatient visits. Finally, extra information was extracted from the database about the fecal continence and defecation pattern during the most recent visit.

### Questionnaire

The survey included questions about the delivery method. It was specifically asked what delivery method was planned, what the considerations were, and what the delivery outcome was. In the case of a vaginal delivery, it was asked whether an episiotomy was performed. Furthermore, questions were asked about surgical history related to CD, the presence of perianal fistulas, and whether symptoms of perianal fistulas or fecal incontinence worsened because of the pregnancies and deliveries. The data from the medical record was used in case of discrepancy with the data extracted from the questionnaire. The current fecal continence was scored using the Vaizey score, with outcomes ranging from 0 (perfect continence) to 24 (completely incontinent).^18^ The Vaizey score is a validated tool consisting of 2 scoring systems with a five-point scale that evaluates the type and frequency of stool loss, flatus incontinence, and impact on quality of life. The full questionnaire and Vaizey score can be found in the supplementary data.

### Statistical analysis

All data were analyzed by a single author to ensure consistency of interpretation. The data from the questionnaire and the medical records were stored in one large database. Descriptive analysis was performed using SPSS (version 23, IBM, 2018). Differences between delivery methods were analyzed using the chi-square test. The threshold for statistical significance was set at *P*<.05.

## Results

### Patients

**Fig. 1:**
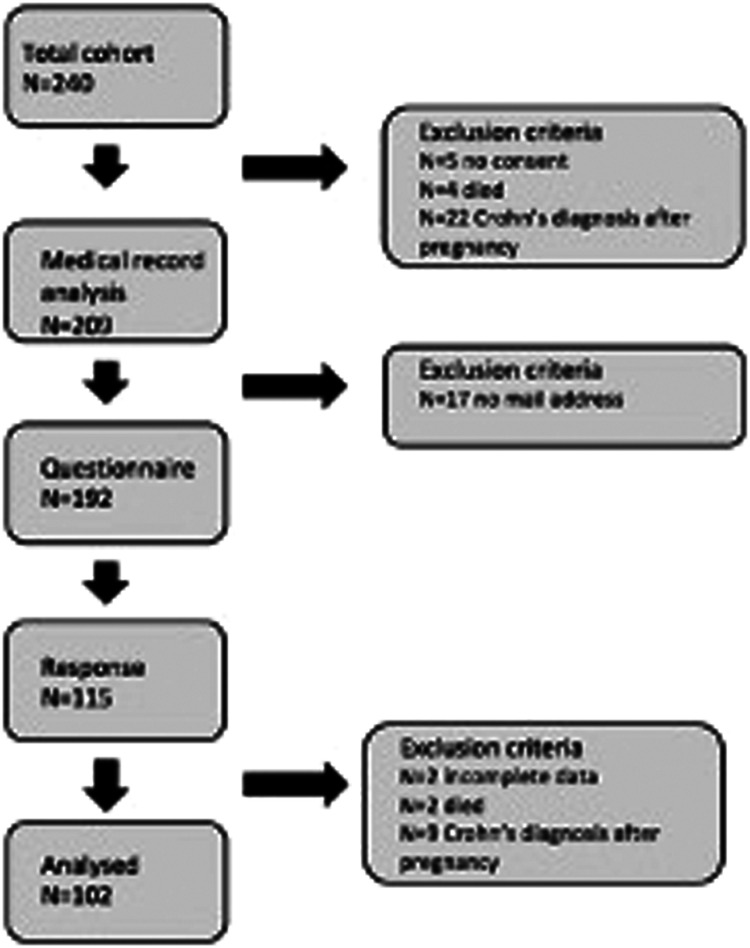
Flow chart inclusion of patients.

The total cohort consisted of 240 patients who met the inclusion criteria. Patients were excluded who were diagnosed with CD after their pregnancy (n=22), who died (n=4), and who did not give consent (n=5). The medical records of 209 patients were analyzed. Distribution of the questionnaire was through email. Patients without email addresses were excluded (n=17). The questionnaire was sent by email to 192 patients, and 115 patients responded (response rate 59,9%). Because of incomplete data (n=2), death of patients (n=2), and extra patients who were diagnosed with CD after the pregnancies (n=9), a total of 102 questionnaires were analyzed, leading to analysis of medical records and questionnaires of 102 patients Figure 1. Furthermore, we found many missing data on pelvic MRIs and delivery features. Therefore, we did not show them in our results.

Schaafsma. Mode of delivery in women with active perianal fistula. Am J Obstet Gynecol Glob Rep 2024.

Patient characteristics are shown in Table 1. Within our cohort, the mean age of women is 50 years. The median interval between the questionnaire and the most recent childbirth is 16 years. In our cohort, 205 deliveries were reported, of which 40 (19.5%) were cesarean deliveries. As reported in the questionnaire, 20% were indicated because of CD. Other reasons were perianal disease (n=5), fetal distress (n=11), maternal health (n=8), and unfeasible normal labor (n=8). A total of 170 vaginal deliveries and 35 cesarean deliveries were advised and planned. Eventually, this resulted in 40 cesarean deliveries and 165 vaginal deliveries.

**Table 1:** Patient characteristics

	Total cohort (n=102)	At least one vaginal delivery (n=84)	Cesarean deliverys (n=18)
Mean age (y), n (range)	50 (27–80)	50 (29–82)	49 (32–64)
Deliveries, n			
Vaginal delivery	165	165	—
Cesarean delivery	40	10	30
Median time till last delivery (y), n (range)	16 (0–55)	17 (0–55)	9 (1–48)
Surgical history, n (%)			
None	47 (45%)	39 (46%)	8 (44%)
Perianal surgery	14 (14%)	12 (14%)	2 (11%)
Ileocecal resection	20 (20%)	13 (15%)	7 (39%)
Subtotal colectomy	7 (7%)	7 (8%)	—
Proctocolectomy	5 (5%)	5 (6%)	—
Multiple small bowel resections	15 (15%)	14 (17%)	1 (6%)
Ostomy formation	2 (2%)	2 (2%)	—
Segmental colonic resection	5 (%%)	3 (4%)	2 (11%)
IPAA	1 (1%)	1 (1%)	—

*IPAA*, ileal pouch anal anastomosis.

### Perianal disease progression

Within the group of women who delivered at least one child vaginally (n=84), 13,1% reported perianal disease progression after their deliveries. In the group of women who delivered only through cesarean delivery (n=18), 22,2% reported perianal disease progression after their deliveries.

### Vaizey score and defecation pattern

To analyze the effect of the mode of delivery on fecal incontinence in patients with perianal CD, women were divided into categories who had either delivered at least one child vaginally (n=84) or had undergone cesarean deliveries during all deliveries (n=18). (Table 2)

**Table 2:** Perianal disease progression and altered fecal continence

Variables	At least 1 vaginal delivery (n=84)	Only cesarean deliveries (n=18)
Perianal disease progression	13.1% (missing data 4)	22.2% (missing data 2)
Altered defecation pattern	25.5%	18.8%
Stool frequency	Median 3.0 (range 1–13), missing data 13	Median 3.5 (range 2–15), missing data 4
Vaizey score	7 (range 0–20)	5 (range 0–12)

Women who had at least one vaginal delivery had a median Vaizey score of 7 (range 0–20). Women who delivered solely through cesarean delivery had a median Vaizey score of 5 (range 0–12). Within the Vaizey score, there was no difference in distribution within the different elements of the score between both groups. Linear regression analysis showed no significant relation between the mode of delivery and fecal incontinence (B, 0.97 [−1,19 to 3,14], *P*=.375). This linear regression analysis was corrected for the possible progression of fecal incontinence with age, using the number of years between the most recent delivery and completion of the questionnaire.

Within the group of women who delivered at least one child vaginally (n=84), 25.5% reported an alteration of fecal continence, the median stool frequency is 3 (range 1–13) within this group. Compared with 18,8% of women who only had cesarean deliveries (n=18), who had a median stool frequency of 3.5 (range 2–15). We performed a chi-square test to compare the results, showing no significant difference between the groups.

## Discussion

### Principal findings

In our study, an overall cesarean delivery rate of 19.5% was observed. 22.5% of the women report a negatively altered defecation pattern after their pregnancies and deliveries. Importantly, no significant relation was found between the alteration of the defecation pattern and the mode of delivery in this study.

After a median follow-up of 15 years, the average Vaizey score within the group of women who delivered vaginally (Vaizey score of 7) seems to be slightly higher than the average Vaizey score of women who have undergone a cesarean (Vaizey score of 5). However, there is no significant relationship between the mode of delivery and the Vaizey score for fecal continence.

### Results in the context of what is known

The cesarean delivery rate is higher than the general population in the Netherlands, where a cesarean delivery rate of 13,6% is seen.^17^ However, the cesarean delivery rate of 19,5% is remarkably lower than reported in other studies focusing on women with CD. Burke et al^15^ report a cesarean delivery rate of 83,3% in women with CD and perianal disease in their medical history. In the Netherlands, the caesarian section rate is lower than that of other countries. We cannot explain these differences differently than patients and doctors being more motivated to proceed with vaginal delivery if not absolutely contraindicated.

An important outcome when considering the mode of delivery in women with CD is the possible worsening of fecal continence. Ong et al^10^ analyzed subjective fecal incontinence after delivery in a survey study of CD patients compared with healthy parous controls. Ong et al^10^ described an incontinence rate of 24% in the CD patients, compared with 2% in the control group. In addition, in a large study among the general Dutch population (n=1259), a median Vaizey score of 11 (range 3–20) was reported.^19^ This implies that, generally speaking, the women in this cohort have better continence than the general population, both in the vaginal delivery group and the cesarean delivery group.

In other studies, it had also been concluded that the occurrence of perianal fistulas was independent of the mode of delivery^13^ and that less perianal disease progression was seen in women who had a vaginal delivery compared with women who had undergone cesarean delivery.^14^

### Clinical implications

It is very relevant to determine the effect of the mode of delivery on perianal disease progression and fecal incontinence, as this gives rise to a serious reduction in the quality of life. The current ECCO guideline discriminates between inactive and active fistulizing perianal disease, which is a subjective and difficult distinction in daily clinical practice. Furthermore, when cesarean delivery is recommended, it is important to consider the fact that in the general population, higher rehospitalization rates are documented after cesarean delivery than spontaneous vaginal delivery.^20^ In addition, it should be noted that a cesarean delivery results in maternal morbidity and increased hospital costs than vaginal deliveries. Furthermore, a CD-related complication of a cesarean delivery is fistula formation in the surgical wound.^20^^,^^21^ Therefore, we suggest more tailored obstetric advice in women with perianal Crohn's disease.

### Research implications

There is little known about the impact of the mode of delivery in women with CD, especially when complicated by perianal disease. Known concerns are the effects on perianal disease progression and the risk of incontinence. To formulate proper scientifically based advice on the mode of delivery, better data registration, such as fistula location and Perianal Disease Activity Index, is needed. Eventually, we propose that the collection of high-quality data could lead to an adjustment of the ECCO guidelines.

### Strengths and limitations

Our study has a few limitations. In some cases, there is a long interval between the childbirth and the administration of the survey (0–55 years), which might lead to recall bias. The reported (in)continence and possible changes in bowel habits related to the delivery should, therefore, be interpreted with caution. In addition, the sample size in the subgroups was small, even though the entire cohort included many patients.

Furthermore, we cannot conclude the effect of fistula location and complexity on fecal incontinence or perianal disease progression based on our data. In our opinion, when treating pregnant patients with perianal CD, fistulas located at the dorsal side of the anus are unlikely to affect and be affected by a vaginal delivery. In addition, it was difficult to obtain information on the activity of the fistulas. This was not properly documented, and often, no MRI was available. Therefore, classifying fistula and their impact is impossible in our study.

## Conclusions

Despite the limitations of this study, we believe that our data suggest that the occurrence of an altered defecation pattern and perianal disease progression is independent of the mode of delivery, both in women with active perianal disease and in women with inactive perianal disease. Therefore, our results question the recommendation of cesarean delivery in case of active perianal disease as described in the ECCO guideline.^3^

The advice on the mode of delivery in women with perianal CD should remain subject to a multidisciplinary approach and should mainly be governed by obstetric indications in consultation with the patient.

## CRediT authorship contribution statement

**Irene J. Schaafsma:** Writing – original draft, Methodology, Investigation. **Froukje J. Hoogenboom:** Writing – review & editing, Supervision, Methodology, Conceptualization. **Gerard Dijkstra:** Supervision. **Jelmer R. Prins:** Writing – review & editing, Methodology. **Marijn C. Visschedijk:** Writing – review & editing, Methodology, Conceptualization.

## Appendix Supplementary materials

Image, application 1

## Footnotes

Supplementary material associated with this article can be found, in the online version, at doi:10.1016/j.xagr.2024.100333.

## References

[1] Caprilli R, Gassull MA, Escher JC (2006). European evidence based consensus on the diagnosis and management of Crohn's disease: special situations. Gut.

[2] Hellers G, Bergstrand O, Ewerth S, Holmström B (1980). Occurrence and outcome after primary treatment of anal fistulae in Crohn's disease. Gut.

[3] Torres J, Chaparro M, Julsgaard M (2023). European Crohn's and colitis guidelines on sexuality, fertility, pregnancy, and lactation. J Crohns Colitis.

[4] Brandt LJ, Estabrook SG, Reinus JF (1995). Results of a survey to evaluate whether vaginal delivery and episiotomy lead to perineal involvement in women with Crohn's disease. Am J Gastroenterol.

[5] Ilnyckyj A, Blanchard JF, Bernstein CN (1998). Perianal Crohn's disease and pregnancy: role of the mode of delivery. Gastroenterology.

[6] Hatch Q, Champagne BJ, Maykel JA (2014). Crohn's disease and pregnancy: the impact of perianal disease on delivery methods and complications. Dis Colon Rectum.

[7] Selinger C, Carey N, Cassere S (2021). Standards for the provision of antenatal care for patients with inflammatory bowel disease: guidance endorsed by the British Society of Gastroenterology and the British Maternal and Fetal Medicine Society. Frontline Gastroenterol.

[8] Nguyen GC, Seow CH, Maxwell C (2016). The Toronto consensus statements for the management of inflammatory bowel disease in pregnancy. Gastroenterology.

[9] Mahadevan U, Robinson C, Bernasko N (2019). Inflammatory bowel disease in pregnancy clinical care pathway: a report from the American Gastroenterological Association IBD parenthood project working group. Gastroenterology.

[10] Ong JP, Edwards GJ, Allison MC (2007). Mode of delivery and risk of fecal incontinence in women with or without inflammatory bowel disease: questionnaire survey. Inflamm Bowel Dis.

[11] Aitola P, Lehto K, Fonsell R, Huhtala H (2010). Prevalence of faecal incontinence in adults aged 30 years or more in general population. Colorectal Dis.

[12] Norton C, Dibley LB, Bassett P (2013). Faecal incontinence in inflammatory bowel disease: associations and effect on quality of life. J Crohns Colitis.

[13] Grouin A, Brochard C, Siproudhis L (2015). Perianal Crohn's disease results in fewer pregnancies but is not exacerbated by vaginal delivery. Dig Liver Dis.

[14] Smink M, Lotgering FK, Albers L, de Jong DJ (2011). Effect of childbirth on the course of Crohn's disease; results from a retrospective cohort study in the Netherlands. BMC Gastroenterol.

[15] Burke KE, Haviland MJ, Hacker MR, Shainker SA, Cheifetz AS (2017). Indications for mode of delivery in pregnant women with inflammatory bowel disease. Inflamm Bowel Dis.

[16] Stichting perinatale registratie Nederland (2009). https://assets.perined.nl/docs/0512b8fb-946d-4520-83c4-7e13ccef2caa.pdf.

[17] Kwee A, Elferink-Stinkens PM, Reuwer PJ, Bruinse HW (2007). Trends in obstetric interventions in the Dutch obstetrical care system in the period 1993-2002. Eur J Obstet Gynecol Reprod Biol.

[18] Vaizey CJ, Carapeti E, Cahill JA, Kamm MA (1999). Prospective comparison of faecal incontinence grading systems. Gut.

[19] van Meegdenburg MM, Meinds RJ, Trzpis M, Broens PMA (2018). Subtypes and symptoms of fecal incontinence in the Dutch population: a cross-sectional study. Int J Colorectal Dis.

[20] Lydon-Rochelle M, Holt VL, Martin DP, Easterling TR (2000). Association between method of delivery and maternal rehospitalization. JAMA.

[21] Liu S, Heaman M, Kramer MS (2002). Length of hospital stay, obstetric conditions at childbirth, and maternal readmission: a population-based cohort study. Am J Obstet Gynecol.

