# Supplementary material for: Vaginal delivery in women with perianal Crohn's disease: why not?
Source: AJOG Glob Rep. 2024 Mar 14;4(2):100333. doi: 10.1016/j.xagr.2024.100333 (PMC11035108; doi:10.1016/j.xagr.2024.100333)
Supplement: Supplementary file 1 [file mmc1.docx]

Supplementary data

Vaizey- Score

|  | Never | Rarely | Sometimes | Weekly | Daily |
| --- | --- | --- | --- | --- | --- |
| Incontinence for solid stool | 0 | 1 | 2 | 3 | 4 |
| Incontinence for liquid stool | 0 | 1 | 2 | 3 | 4 |
| Incontinence for gas | 0 | 1 | 2 | 3 | 4 |
|  |  |  |  | No | Yes |
| Need to wear a pad or plug |  |  |  | 0 | 2 |
| Taking constipating medications |  |  |  | 0 | 2 |
| Lack of ability to defer defecation for 15 min |  |  |  | 0 | 4 |

Questionnaire

You have been selected for this questionnaire as you are familiar with Crohn’s disease, have given birth to at least one child and have (had) perianal disease, according to our data.

1. Have you (had) perianal fistulas? YES / NO

2. Have you undergone any bowel surgery? YES / NO

| YES / NO | Year? |
| --- | --- |
|  |  |
|  |  |
|  |  |
|  |  |
|  |  |

Did you have a bowel resection? Which year?

Did you get a stoma? Which year?

Did you get an ileo-anal pouch reconstruction? Which year?

Did you undergo anal surgery? Which year?

Did you undergo surgery for perianal fistulas? Which year?

3. Did you get children? YES / NO

4. What was the planned mode of delivery? (Circle the applicable answer)

Child 1 Vaginal delivery Cesarean delivery

Child 2 Vaginal delivery Cesarean delivery

Child 3 Vaginal delivery Cesarean delivery

Child 4 Vaginal delivery Cesarean delivery

5. What was the mode of delivery? (Circle the applicable answer):

(Assisted vaginal delivery comprises a vacuum- or forceps delivery. CS is cesarean delivery)

Child 1 Year of birth… Vaginal delivery, Assisted vaginal delivery, Emergency CS, Scheduled CS

Child 2 Year of birth… Vaginal delivery, Assisted vaginal delivery, Emergency CS, Scheduled CS

Child 3 Year of birth… Vaginal delivery, Assisted vaginal delivery, Emergency CS, Scheduled CS

Child 4 Year of birth… Vaginal delivery, Assisted vaginal delivery, Emergency CS, Scheduled CS

6. Did you undergo an episiotomy? YES/NO/I DON’T KNOW

7. When your first delivery was a cesarean delivery, why was it decided?

a. Recommended because of my own health (e.g. high blood pressure)

b. Recommended because of my Crohn’s disease

c. Recommended because of active perianal fistulas

d. Recommended because of my perianal fistulas in the past

e. Recommended because of the health of the unborn baby

f. The vaginal delivery did not proceed, so I needed an emergency CS

g. I personally preferred a ceasearean section

h. I do not know

8. Did you have a cesarean delivery during other deliveries YES / NO

| Amount: |  |
| --- | --- |

9. How many?

10. Why was this decided?

a. Recommended because of my own health (e.g. high blood pressure)

b. Recommended because of my Crohn’s disease

c. Recommended because of active perianal fistulas

d. Recommended because of my perianal fistulas in the past

e. Recommended because of the health of the unborn baby

f. The vaginal delivery did not proceed, so I needed an emergency CS

g. I personally preferred a ceasearean section

h. I do not know

11. When you personally preferred a cesarean delivery, was this because of your Crohn’s disease? YES / NO

Or was this because of your perianal fistulas? YES / NO

12. Did you have problems with faecal continence prior to your first pregnancy and delivery? (need of clean underwear, not being able to defer defecation for 15 minutes, excessive gas, the need for incontinence-pads etc.)

YES / NO

13. Were you afraid of excessive gas or soiling in your underwear prior to your pregnancies?

YES / NO

14. Can you please score your current faecal continence below?

I am incontinent for solid stool Never Rarely Sometimes Usually Always

I am incontinent for liquid stool Never Rarely Sometimes Usually Always

I am incontinent for gas Never Rarely Sometimes Usually Always

I alter my lifestyl Never Rarely Sometimes Usually Always

I need to wear pads in my underwear Never Rarely Sometimes Usually Always

I take constipating medication YES NO

I am not able to defer the defecation for 15 minutes YES NO

15. How often do you defecate on average (per day)?

16. Was your defecation pattern/faecal continence altered after your pregnancies? YES / NO

17. Was your defecation pattern or faecal continence specifically altered after your…

| First pregnancy/delivery? | YES/NO/I DON’T KNOW |
| --- | --- |
| Second pregnancy/delivery? | YES/NO/I DON’T KNOW |
| Third pregnancy/delivery? | YES/NO/I DON’T KNOW |
| Fourth pregnancy/delivery? | YES/NO/I DON’T KNOW |

18. Did you experience problems with perianal fistulas prior to your pregnancy?

Child 1 YES / NO

Child 2 YES / NO

Child 3 YES / NO

Child 4 YES / NO

19. Did you experience problems with perianal fistulas during your pregnancy?

Child 1 YES / NO

Child 2 YES / NO

Child 3 YES / NO

Child 4 YES / NO

20. Did you experience more problems with perianal fistulas after your delivery?

Child 1 YES / NO

Child 2 YES / NO

Child 3 YES / NO

Child 4 YES / NO

21. In case you were not familiar with perianal fistulas, did you get perianal fistulas after the delivery?

Child 1 YES / NO

Child 2 YES / NO

Child 3 YES / NO

Child 4 YES / NO
